# Rapid and Detailed Characterization of Transgene Insertion Sites in Genetically Modified Plants via Nanopore Sequencing

**DOI:** 10.3389/fpls.2020.602313

**Published:** 2021-02-04

**Authors:** Paula A. Giraldo, Hiroshi Shinozuka, German C. Spangenberg, Kevin F. Smith, Noel O. I. Cogan

**Affiliations:** ^1^Faculty of Veterinary and Agricultural Sciences, The University of Melbourne, Parkville, VIC, Australia; ^2^Agriculture Victoria Research, AgriBio, The Centre for AgriBioscience, Bundoora, VIC, Australia; ^3^AgriBio, The Centre for AgriBioscience, School of Applied Systems Biology, La Trobe University, Bundoora, VIC, Australia; ^4^Agriculture Victoria Research, Hamilton, VIC, Australia

**Keywords:** sequence, GMO, ryegrass, canola, clover, transgenic, MinION

## Abstract

Molecular characterization of genetically modified plants can provide crucial information for the development of detection and identification methods, to comply with traceability, and labeling requirements prior to commercialization. Detailed description of the genetic modification was previously a challenging step in the safety assessment, since it required the use of laborious and time-consuming techniques. In this study an accurate, simple, and fast method was developed for molecular characterization of genetically modified (GM) plants, following a user-friendly workflow for researchers with limited bioinformatic capabilities. Three GM events from a diverse array of crop species—perennial ryegrass, white clover, and canola—were used to test the approach that exploits long-read sequencing by the MinION device, from Oxford Nanopore Technologies. The method delivered a higher degree of resolution of the transgenic events within the host genome than has previously been possible with the standard Illumina short-range sequencing strategies. The flanking sequences, copy number, and presence of backbone sequences, and overall transgene insertion structure were determined for each of the plant genomes, with the additional identification of moderate-sized secondary insertions that would have previously been missed. The proposed workflow takes only about 1 week from DNA extraction to analyzed result, and the method will complement the existing approaches for molecular characterization of GM plants, since it makes the process faster, simpler, and more cost-effective.

## Introduction

Implementation of plant biotechnology has enabled targeted alterations of crop traits through genetic transformation. The insertion of a specific transgenic element, containing a desired gene, into the host genome is the most common application of this technology to date (Fraiture et al., 2017). Safety assessments of new transgenic plants are essential in order to identify potential threats to humans, animals, and the environment. The first step in any risk evaluation of genetically modified (GM) plants, prior to their approval for release into the market, is the molecular characterization of the transgene(s). Such characterization must include identification of the locus/loci of the genetic modification, detailing the flanking genomic regions, copy number of the inserted transgene expression cassette, and endogenous host gene interruptions by the transgenic DNA (Schouten et al., 2017).

Cultivation of biotech (GM) crops is expanding rapidly, triggering the need for accurate and cost-effective molecular characterization methods (Li et al., 2017). Copy number determination of the transgenic event is the first parameter to be considered, as no transformation method can fully control the number of transgene insertions into the host genome (Tiwari and Singh, 2018). The most universally accepted techniques for molecular characterization of new transgenic events have been DNA blot analysis, along with polymerase chain reaction (PCR) (Li et al., 2017). These methods can attempt to determine transgene copy number but fail to provide a detailed structure of the insertion.

DNA sequencing approaches have been used to determine the insertion site, flanking regions, and any possible endogenous gene interruption caused by the transgenic insert. The traditional and gold standard method for this purpose has been Sanger sequencing. Despite the robustness of Sanger sequencing, its implementation requires several steps that make it a time-consuming and expensive method that can struggle to accurately sequence complex regions of the genomes (Guttikonda et al., 2016). Hence, during the last decade second generation sequencing (SGS, also called next generation sequencing) has been proposed as a cost-effective and rapid option for routine sequencing (Kovalic et al., 2012; Yang et al., 2013; Pauwels et al., 2015; Arulandhu et al., 2016). Although SGS, offers high-throughput, scalability, and time effectiveness (Kovalic et al., 2012; Wahler et al., 2013; Yang et al., 2013; Guttikonda et al., 2016), its short-read length nature makes the bioinformatic analysis highly challenging, due to the high percentage of ambiguous or incorrectly mapped reads (Park et al., 2017). A combination of PCR-based genome walking methods, to enrich the target DNA region, with sequencing strategies is gaining popularity for routine molecular characterization of transgenic events (Li et al., 2017). However, such enrichment methods are also associated with short DNA fragments, failing to characterize unintended insertions. Such a drawback is particularly problematic in plants with complex insertions, including endogenous genes or promoters, GC rich, and/or genomes with a high content of repetitive regions.

To overcome the assembly issues with SGS, recently third-generation sequencing (TGS, also called single-molecule sequencing) has emerged. TGS has increased read lengths up to hundreds of kilobases, as well as reducing the sequencing time (Jain et al., 2016). The ability to increase read lengths can facilitate GM molecular characterizations, potentially solving alignment problems caused by repetitive and low complexity regions of a genome (Istace et al., 2017). Currently, there are two TGS platforms commercially available, Pacific Biosciences (PacBio) and Oxford Nanopore Technologies (ONT). The potential benefits of the MinION ONT include long reads that may exceed 200 kb, real-time data delivery, low cost, and portability (Magi et al., 2017).

Recently, ONT sequencing has been coupled with DNA walking methods to detect un-authorized GM organisms (Fraiture et al., 2018) and to identify transgenic alleles in soybean, when breeding to introgress them into other cultivars (Li et al., 2019). However, this approach can fail to determine features such as copy number, deletions or unintended insertions, and the use of primer walking methods to target specific regions, limiting the size of DNA fragments. Low coverage whole-genome shotgun sequencing can be the fastest way to identify transgenic insertions, due to the reduction in sequencing cost and incremental advances of data yields and accuracy of the ONT flowcells (Michael et al., 2018). This approach is particularly effective when the transgene integrates into complex repetitive regions of the genome, or when the transgene is cisgenic in nature and an endogenous copy of the same sequence is already present in the target genome, making walking strategies highly challenging. The major challenge in nanopore sequencing is the error rate (5–15%; Luo et al., 2019), which is particularly important when detecting single-nucleotide polymorphisms (SNPs). However, improvements in the flowcells chemistry (R9 replacing the previous R7 version and the release of the R10 pore) and sequence analysis tools are constantly providing higher accuracy (Senol-Cali et al., 2019). Another challenge to successful use of TGS technologies in the molecular characterization of transgenic plants is the data analysis step, which requires bioinformatic pipelines and fit-for-purpose bioinformatic libraries (Li et al., 2017).

In this article, we demonstrate how one can rapidly and inexpensively complete the full molecular characterization of a transgenic event by using a single ONT MinION flowcell. Three transgenic crops, spanning mono- and dicotyledonous species, with different genome sizes, complexity, and availability of reference genomes, were used as examples to show the utility of this methodology. The purpose of the present shotgun (random) whole genome sequencing is to identify reads that encompass the genome-transgene boundary, which can describe the transgene integration site on the genome and copy number. Due to the evenness of genome coverage from the ONT technology (Malmberg et al., 2019), an overall lower coverage (5–10x) could be sufficient to achieve satisfactory resolution of the transgene, as long as the DNA extracted is of sufficient length and generates long enough sequence reads to provide the resolution required. The major contribution of this method is the user-friendly data analysis workflow, which is particularly useful for researchers with limited bioinformatic resources. Recently, different studies used ONT to sequence transgenic events using different approaches (Nicholls et al., 2019; Boutigny et al., 2020; Voorhuijzen et al., 2020). However, to the best of our knowledge this is the first time that ONT has been applied to fully identify and completely characterize plant GM events at the molecular level in an unbiased untargeted manner, which will set the basis for transgene event sequencing into the future.

## Materials and Methods

Three GM crops, canola, white clover, and perennial ryegrass, were used as examples for the approach using the MinION, from ONT, for molecular characterization of GM crops using a common analytical process. The sequences of the complete vectors were known, and a graphic description of the material used for the transformation is shown in Supplementary Figure S1.

### Plant Material and Transformation

Canola, an allopolyploid crop with a 1,130 Mb genome size (Chalhoub et al., 2014), was transformed with the ETIP vector using non-canonical zinc finger nucleases (ZFNs) for targeted modification of plant genomes (Supplementary Figure S1A), and its transformation is described in Cogan et al. (2014). Additionally, white clover, an allotetraploid crop with 1,093 mb genome size (Bennett and Leitch, 2011), was transformed using *Agrobacterium tumefaciens* (denominated pCLV000032 vector) containing three new traits for delayed leaf senescence, aluminum tolerance, and resistant to AMV (Alfalfa mosaic virus), and the *hph* gene (pDOI000080 vector) as a selectable marker, was also used (Supplementary Figure S1B). Detailed description of the transformation with the multi-trait construct and selectable marker transgenes is reported in Narancio (2018). Finally, perennial ryegrass, which has a diploid genome with 2,700 Mb in size (Kopecky et al., 2017), was bombarded via biolistics with LpOs6g0607800 RNAi (glutamate receptor pGRA000120) construct (Supplementary Figure S1C). The glutamate receptor is a potential male candidate gene for the *S* locus that partly controls the ryegrass self-incompatibility process and pollen recognition (Spangenberg et al., 2019; Shinozuka et al., 2019).

### DNA Extraction, Concentration, and Purity

DNA was extracted using a modified version of the high molecular weight genomic DNA extraction from plant species (Vaillancourt and Buell, 2019). In summary, after disruption, plant samples were digested and genomic DNA was purified in three separate steps: G2 lysis buffer (QIAGEN, Hilden, Germany), RNase, and proteinase K. Then the samples were transferred into equilibrated QIAGEN Genomic-tip 100/G columns following the manufacturer’s instructions (QIAGEN, Hilden, Germany), washed, and the DNA eluted. Finally, the DNA was washed with isopropanol and ethanol and resuspended in TE buffer.

**FIGURE 1:**
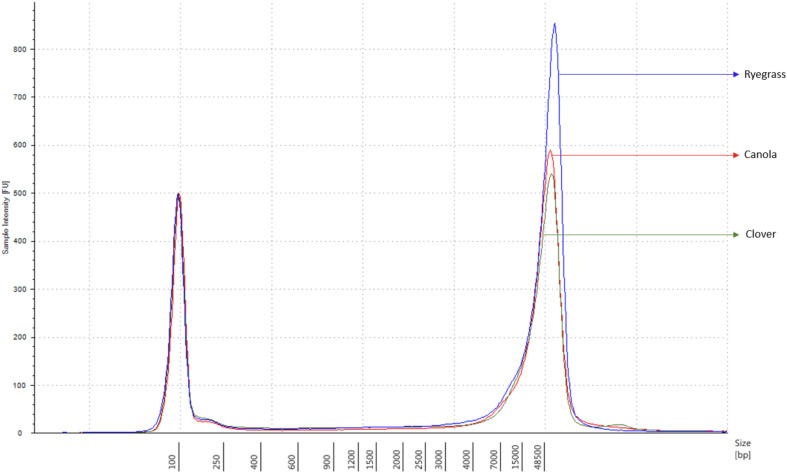
Sample comparison of each extracted genomic DNA from canola, clover, and ryegrass, using the function of the 2200 TapeStation software with a ladder of 60 kbp maximum measurement.

Each sample concentration was measured using the Qubit dsDNA HS assay kits and a Qubit 3.0 Fluorometer (Invitrogen^TM^, Thermo Fisher Brand, CA, United States), according to the manufacturer’s instructions. DNA quality was measured with the NanoDrop 1000 UV-vis spectrophotometer (ThermoFisher) using the A260/A280 and A260/A230 ratios, and the DNA size was analyzed using the Genomic DNA ScreenTape, through the Agilent 2200 TapeStation system (Agilent Technologies, Santa Clara, CA, United States), according to the manufacturer’s instructions. Samples with a minimum amount of 1.5 μg of genomic DNA in 47 μl of distilled water, A260/A280 ratio of 1.8 ± 0.1, A260/A230 ratio of 2.0 ± 0.1, and DNA length above 60 kbp were used for sequencing purposes (Figure 1).

### Library Preparation and Sequencing

Each sequencing library was prepared from 1.5 μg of high molecular weight genomic DNA using the 1D Genomic DNA by ligation protocol (SQK-LSK109; ONT, Oxford, United Kingdom), according to the manufacturer’s instructions. DNA fragmentation was not performed, to maintain the integrity of the high molecular weight DNA. Briefly, the DNA was repaired and blunt-ended, followed by an AMPure XP bead clean-up. Then, the DNA was adapter-ligated and cleaned again, to finalize with a library priming and loading. The prepared library was loaded into a MinION Mk1B device using 106 R9 flowcells (ONT, Oxford, United Kingdom). In total, three flowcells were used, one for each crop, and each sequencing run was performed for 48 h using live base calling in the MinKNOW software version 1.4-1.13.1 (ONT). The quality control assessment of the raw generated data, in the fastq format, was analyzed using python (version 3.6.4) and albacore (version 2.3.4).

### Sequencing Data Analysis

Analysis of the sequence dataset was performed using local BLAST (version 2.2.26) searches. Although, simple BLAST analyses can be easily performed online, and when using large data sets it is more practical to use a local version. The BLAST program was downloaded from http://www.ncbi.nlm.nih.gov/guide/data-software/. Once installed the local BLAST program was run using a command line interface, as described in GitHub^1^. The software used to perform the base calling (MinKNOW) provides the sequencing reads in FASTQ format, so they were transformed into FASTA using the Seqtk tool as described in GitHub^2^. Then, all nanopore sequencing reads for each sample were converted into a BLAST database, and each transgenic vector sequence, including the transgene construct and backbone sequence, was used as the query in the analysis, following the GitHub local BLAST instructions. An *e*-value threshold 0 was used as a search option to filter reads with higher similarity (imposed as e < 1e-200). Blast results were pasted into an excel file for visual inspection, as described in Supplementary Table S1. Selected reads were retained and isolated, using seqtk (subseq; see footnote 2) tools. Reads were manually mapped and visually checked using the software Sequencher (version 5.4.6; Gene Codes, Ann Arbour, MI, United States).

To identify the exact location of the transgenic inserts within the host genome, a further sequence homology analysis using BLAST was performed. In this instance, the database was generated from each crop reference genome (full, relative, or draft genome), and the query sequences were all reads that hit each transgenic vector from the previous step. Due to the cisgenic nature of several elements present in the transgenic vectors, sequence reads matching only endogenous elements with endogenous flanking regions were discarded. The analysis was also filtered by *e*-value, so only reads with *e*-value threshold 0 were selected.

The reference genome used for *Brassica napus* (canola) is reported in Chalhoub et al. (2014) and in the absence of a *Trifolium repens* (white clover) reference, the *Trifolium pratense* genome published in De Vega et al. (2015) was used. For *Perennial ryegrass* there is not a full reference genome available, so three drafts were used (Byrne et al., 2015; Shinozuka et al., 2016; Velmurugan et al., 2016).

## Results

**FIGURE 2:**
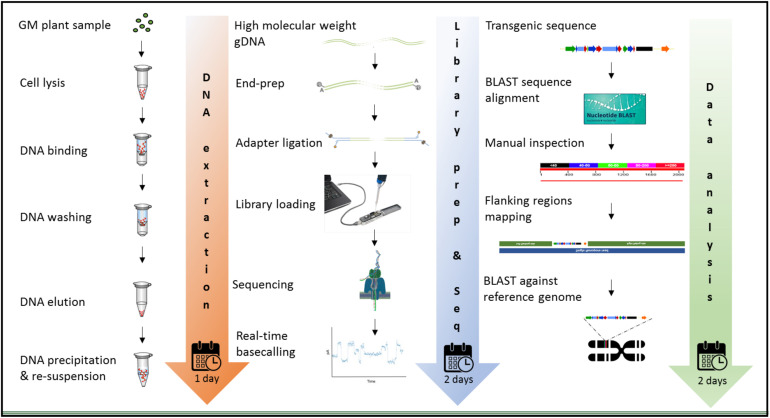
Workflow for the molecular characterization of genetically modified plants, using the MinION device of ONT. Graphic illustration from DNA extraction **(left)**, library preparation and sequencing **(middle)**, and data analysis **(right)**.

The MinION device from ONT was tested for molecular characterization of GM crops using a common analytical process. Three crops contrasting in genome size, complexity of integration, available genome resources, and transformation method were selected. Canola (*Brassica napus* L.) has a full reference genome publicly available, that is 1,130 mb in size, and was transformed with a transgenic construct of 10,648 bases with a GC content of 46.5%. White clover (*Trifolium repens* L.) has a 1,093 mb genome and was transformed with two vectors; a multi-trait construct of 8,604 bases in size, 55.19% of which were endogenous elements (Supplementary Figure S1B) and 45.8% GC content, and a selectable marker vector containing hygromycin phosphotransferase gene (*hph*) of 3,348 bases in size and 53.4% of GC content. Although white clover does not have a reference genome, *Trifolium pratense*, a close relative does have a genome sequence, which was used. In contrast, perennial ryegrass (*Lolium perenne* L.) had the largest genome size of 2,700 mb among the three species studied, and it does not have any reference genome or close relative genome to be used as a proxy, so three published drafts were used in this study. Perennial ryegrass was transformed using a biolistic method with a transgenic cassette of 6,401 in size, where 15.12% of the genomic elements are endogenous (Supplementary Figure S1C) and has 47.6% of GC content. Graphic description of the entire workflow is shown in Figure 2.

The extraction of purified high molecular weight DNA is critical in the proposed workflow (Figure 2). The method followed a modified version of the recommended protocol from ONT, for high molecular weight genomic DNA extraction from plant leaves. Sufficient yield for library preparation was achieved from all samples. DNA purity, assessed by absorbance ratios 260/280 nm and 260/230 nm, was measured with values obtained of 1,81 and 2,12 for canola; 1,85 and 2,15 for clover, and 1,87 and 2,08 for ryegrass respectively. DNA fragment length of all samples was assessed by microfluidic electrophoresis, and all samples presented an average fragment length of c. 50 kbp (Figure 1). For each library preparation a total of 1.5 μg of genomic DNA was used as input and the final sequencing libraries, ranged in mass from 559 to 696 ng. Before priming and loading the library into each MinION flowcell, a platform-QC protocol was executed, the number of pores available were 1402, 1531, and 1581 for canola, clover, and ryegrass flow cells respectively. Finally, each library was primed, loaded into each flow-cell, and the sequencing run was started using the MinKNOW software.

### Sequencing Data Generation

Each sample was independently prepared and sequenced on a single MinION flowcell. One hour after sequencing had been initiated, each experiment was visually checked and the MinKNOW metrics were exported. The number of pores sequencing were 368, 274, and 395 for canola, white clover, and perennial ryegrass respectively. The overall read length was longer for canola and ryegrass libraries (10.6 and 11.9 kb), compared to that of clover (1.6 kb) (Supplementary Figure S2).

Once each sequencing run finished (after 48 h), the quality of all raw data was analyzed using the NanoPlot software (Supplementary Figure S3). The total gigabases generated for canola, clover, and ryegrass were 10.4, 7.3, and 8.7, while the total reads generated were 986,303, 4,600,714, and 728,000 respectively. The N50 read length was 24.2, 4.5, and 22.1 kb, and the maximum read lengths were 196, 130, and 173 kb for canola, clover, and ryegrass respectively. Overall, canola and ryegrass samples provided large numbers of long reads with a median read length of 7.3 and 4.9 kb, while clover only presented a median of 0.5 kb. However, the inconvenience of much shorter reads for clover is partially compensated by a much higher number of reads generated, which are 5–6 times more than with canola and ryegrass (Table 1 and Supplementary Figure S3).

**TABLE 1:** Nanopore sequence read lengths and metrics for the three sequencing experiments.

QC parameters	Canola	Clover	Ryegrass
Mean read length (base)	10,577.7	1,584.6	11,923.0
Mean read quality (Q score)	10.0	9.7	9.8
Median read length (base)	4,942.0	577.0	7,321.0
Median real quality (Q score)	10.1	9.7	9.9
Number of reads	986,303.0	4,600,714.0	728,000.0
Total bases	10,432,863,768.0	7,290,150,516.0	8,679,914,588.0
Maximum length (base)	196,540.0	129,999.0	172,891.0

### Detection of Transgenic Insertions

The canola sample was transformed by a single engineered transgene integration platform (ETIP) vector (Supplementary Figure S1A), and during the BLAST search, 34 sequence reads with identified similarity lengths to the vector ranging from 471 to 11,591 bases were selected. The sequence homology analysis with BLAST for the multi-trait construct in clover identified 65 sequence reads with sequence similarity with lengths between 710 and 6,876 bases, while there were 14 sequences for *hph* with read lengths between 997 and 3,686 bases. Ryegrass presented eight sequences with sequence similarity to the relevant vector of between 497 and 5,992 bases.

### Location of Transgenic Vectors

Sequence reads may contain repetitive elements. Therefore, it was necessary to carefully examine each output of the analysis with specific attention to the length of the alignment as well as the location, looking for commonality in the chromosomal position of the longest sequence matches, while discounting small alignments to the array of repeats across the genome.

**FIGURE 3:**
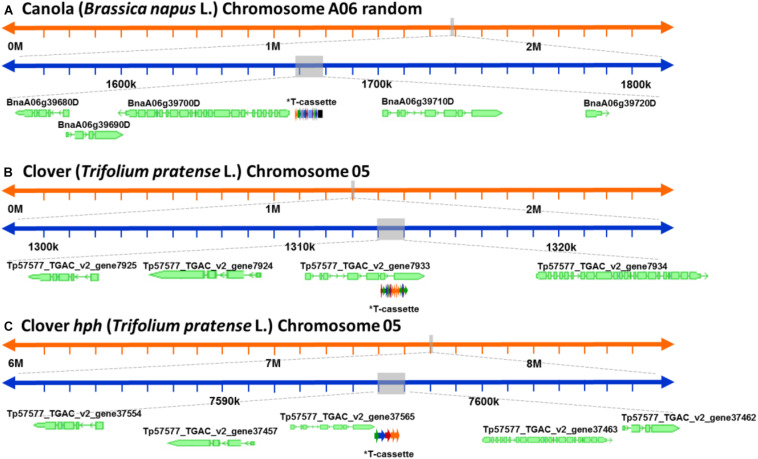
Schematic representation of the genomic location of the genetically modified plants. **(A)** Genomic location of the ETIP transgene on canola’s chromosome 6 random. **(B)** Predicted genomic location of the multi-trait construct on clover’s chromosome 5. **(C)** Predicted genomic location of the hygromycin resistance gene (*hph*) on clover’s chromosome 2. *T-cassette: transgenic cassette.

**FIGURE 4:**
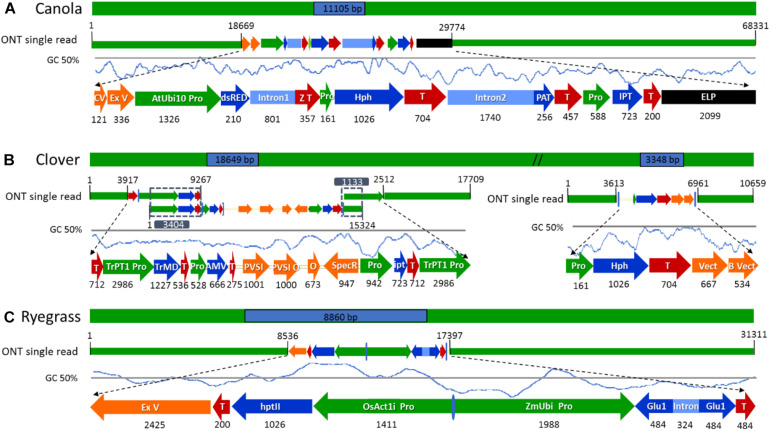
Schematic diagram of the longest nanopore sequence reads describing the complete insertion site, flanking sequences, and transgene rearrangements in the selected genetically modified plants. **(A)** ETIP transgene inserted into canola’s genome. **(B)** Multi-trait construct and the hygromycin resistance gene (*hph*) inserted into clover’s genome. **(C)** Glutamate transgene inserted into ryegrass’s genome. All numeric values are in base pairs (bp).

After a detailed check of all the reads, 18 canola reads were discarded because the read length was too short (less than 1.000 bases), leaving only 16 reads of the initial 34. The longest 12 reads also had endogenous flanking sequence that aligned to chromosome A06 random. The transgenic sequence location within chromosome A06 random was from 1,675 to 1,689 kbp according to Ensembl Genome Browser^3^, as shown in Figure 3A. Examination of the BLAST results also identified, as expected, the homologous region within the allotetraploid species as C07 at c. 32.1 mbp. The sequence similarity at this location had a higher degree of interruptions, and as a result had a lower contiguity that was reflected with significantly lower score values in the analysis. Sequence matches at the C07 homologous region did not exceed 5 kbp, while the longest contiguous sequence match at A06 random exceeded 15 kbp. The insertion of the ETIP transgene into the chromosome location contained no additional unintended DNA sequences in the 5′ side, where the engineered landing pad (ELP) finished. However, in the 3′ side there was 457 bp of backbone between the canola endogenous sequence and the AtUbi10 promoter. The backbone sequences corresponded to CV and ExV (Figure 4A), a Cloning vector sequence (CV) and an expression vector (ExV), to increase the plasmid protein expression level. The transgene was located in an intergenic region between the genes BnaA06g39700D and BnaA06g39710D and did not disrupt either of them (Figure 3A). Additionally, the remaining four reads that did not align to chromosome A06 random were all located in chromosome C01 from 3,897 to 3,900 kbp. These reads identified an insertion of 680 bp of the transgenic cassette that corresponded to a fragment of the AtORF1 terminator and the intron 2 (Supplementary Figure S1A).

For clover relating to the inserted multi-trait construct, 59 from the initial 65 reads were discarded because the read length was too short, leaving 21 reads containing the transgene that matched the *Trifolium pratense* genome. All reads matched chromosome 5 from 13,124 to 13,140 kbp according to Ensembl Genome Browser (see footnote 3), which corresponded to the transgene integration (full transgenic cassette including backbone and double integration of two T-DNA elements). The transgene was located in an intron of the Tp57577_TGAC_v2_gene7933, which encodes for a 50S ribosomal protein l30-like (Figure 3B). Related to the *hph* transgene, there were 14 reads that matched the hygromycin resistance gene (*hph*), however only the 2 longest reads also had endogenous sequence that matched chromosome 5 from 7,600 to 7,601 kbp (see footnote 3), while the remaining 12 smaller reads were considered too short to be useful. The *hph* transgene was located at an intergeneric region between the Tp57577_TGAC_v2_gene37565 and gene37463, without disrupting either of these genes (Figure 3C).

For ryegrass there is not a reference genome published, and neither a close relative that can be used as a reference, so the exact chromosomal location of the transgene could not be elucidated with the available resources. From the initial eight reads found in the transgenic insertion BLAST, three were discarded after checking read lengths. Then using the BLAST analysis to a collection of draft genome sequences, it is believed that the transgene had only inserted at a single scaffold, as from the five selected reads, four of them matched the same scaffold from one of the draft genomes (Velmurugan et al., 2016). The scaffold identified is 22,641 bp in length and it is likely to be located at a repetitive region, since a BLAST search identified some small fragments of *Brachypodium distachyon* and *Triticum aestivum*.

### Insertion Site and Flanking Regions Confirmation

The subset of sequence reads obtained from the initial BLAST to each vector that were too short to hit the reference genome in the second BLAST (18 reads for canola, 59 + 12 reads from clover, and 3 reads from ryegrass) were used to map both transgenic insertions and flanking regions and confirm the accuracy of the previous steps. Once all transgenic insertions and chromosomal locations were confirmed, the longest read that hit each vector was selected for schematic purposes. In canola a 68.3 kbp-read was selected as the longest sequence with the ETIP vector centrally located, presenting a mismatch rate between the read and vector reference of 3.47%. The selection of such a read allowed the identification of 38.6 kbp of canola endogenous sequence in the 5′ flanking side and 18.7 kbp in the 3′ (Figure 4A).

The *hph* vector containing the *Hygromycin-B-phosphotransferase* selectable marker was detected in the middle of a 10.7 kbp-read with 1.8% mismatch rate between the read and vector reference, leaving 5.1 kbp of clover endogenous sequence in the 5′ and 3.6 kbp in the 3′ flanking side (Figure 4B). A single sequence read with the complete multi-trait construct (9.4 kb) on chromosome 5 was not generated. All sequence reads identified were then used to reconstruct the insertion that have three genes for delayed leaf senescence (IPT gene), aluminum tolerance (TrMDHb gene), and Alfalfa mosaic virus resistance (AMV-CP gene). From the detailed analysis and reconstruction of the insertion, it was identified that the entire transgene cassette, including backbone sequence, had inserted along with a second copy of the OCS terminator and TrPT1 promoter, extending the transgenic insertion to 18.6 kbp. The three longest reads with 9.2, 15.3, and 17.7 kb in length were mapped from 5′ to 3′, with 3.4 and 1.1 kbp overlap respectably to illustrate the insertion (Figure 4B). In ryegrass the transgene, containing the glutamate receptor gene to control ryegrass self-incompatibility, was found in the middle of a 31.3 kb read with 4.2% mismatch rate between the read and vector reference. Additionally, a sequence of 2.4 kbp in length of ExV in the 3′ was identified leaving 8.5 kbp of endogenous sequence in the 5′ and 13.9 kbp in the 3′ flanking side (Figure 4C).

## Discussion

Molecular characterization of new transgenic plants is a prerequisite before commercialization, as recombinant DNA can be randomly inserted into the host genome. Identification of genomic features generated by transgene integrations, including insertion site, presence of backbone, repeated, or rearranged sequences, and copy number are crucial from scientific and regulatory perspectives. Identification of those features would facilitate the understanding of the changes made to the plant and the likely consequences (Jupe et al., 2019). The presence of random insertion(s) can cause host gene interruptions that could have unintended results, or the integration of multiple transgenic sequences in one or more chromosomal locations, which affects the stability of the trait triggering transgene silencing (Tiwari and Singh, 2018).

In this study, nanopore reads between 9.2 to 68.3 kb in length allowed the unequivocal characterization of the transgene insertion sites, flanking regions, and location in the host genome without the requirement of a *de novo* assembly step. The perceived high error rates in ONT sequence reads have proved not to be a hinderance to correctly identify the specific transgene location in any of the species tested, including the highly duplicated complex canola genome. The error rate of ONT and the similarity of the transgenes used in this study to each reference genome make it difficult to provide fixed guidelines on the level of resolution of a minimum detection threshold. Malmberg et al. (2019) identified in canola, due to the close genome similarity between the A and C subgenomes, that many illumina SGS sequence reads misaligned in the genome in small localized areas. However, when long ONT reads were generated, they correctly aligned to the specific sub-genome within the allotetraploid, due to the presence of multiple variants within the larger area characterized. ONT sequence reads, despite the higher error rate compared to SGS, are more able to be accurately resolved than SGS, even within highly complex polyploids with highly similar sub-genomes.

The characterization of three contrasting GM plants was achieved using a single MinION flowcell (with 1400–1600 pores) per plant in the present study. The workflow described here is broadly applicable for the vast majority of plant species and all transgene insertions. However, for crops with large genome sizes, such as wheat (genome size estimated at c.14.5 Gb; Appels et al., 2018), more than one MinION flowcell may be required or the use of higher output devices such as the PromethION (also from ONT) could be more effective. With the requirement of increased confidence for some transgenic events, increased sequence read depths may be required to exclude the possibility of missing an insertion. However, the work presented has extensively characterized the known transgene events and identified additional insertions and complexity that were previously unknown, supporting the recommendation of 5-10x genome coverage for initial screening purposes.

Hitherto, the combination of different existing techniques allowed the identification of transgene copy number and insertion site, but molecular tools failed to comprehensively describe the “whole picture.” The lack of comprehensive characterization was due to the techniques available that targeted parts of the transgene (amplicons between 200 bases and 4 kb), usually short in length, to deduce the presence or absence of the entire vector. The optimal transformation method would integrate the gene of interest into the host genome in a single copy. However, during the transformation process, transgenic DNA can be inserted into the host genome at random (biolistic transformation) or induced locations of double strand breaks (*Agrobacterium* transformation) (Tzfira et al., 2003). Additionally, backbone sequences are frequently integrated along with the transgenic sequence, resulting in chromosomal rearrangement. Therefore, a high percentage of transformed plants deviate from the ideal, containing only part or concatenated fragments of transgenic DNA, which is often underestimated (Gelvin, 2017).

Several publications using different molecular tools have reflected such phenomena. For instance, the initial molecular characterization of soybean event GTS40-3-2 using PCR found a single copy of the cassette (Padgette et al., 1995). However, later research identified the insertion of two unintended segments of the transgene (Product Safety Center, 2000) and unexpected rearrangements of endogenous DNA, causing the amendment of its molecular characterization (Windels et al., 2001). Recently, Nicholls et al. (2019) used the nanopore sequencing technology to detect the site of integration of a fluorescent reporter gene in mouse germ lines, which also allowed the identification of *E. coli* DNA contamination integrated within the transgene. Similarly, the isolation and comparison of the longer reads that mapped to each of the transgenic insertions evaluated in this study allowed the identification of transgenic DNA rearrangements and backbone sequences, which are important features for long term stability of transgene expression (Tiwari and Singh, 2018).

The identification of an unintended insertion of 680 bp transgenic cassette in canola chromosome C01 corresponded to the Open Reading Frame 1 (AtORF1) from *Agrobacterium tumefaciens* and intron 2 from *Arabidopsis thaliana* 4-coumaryl-CoA synthase were attained. Unexpected insertions of backbone sequences, usually associated with the integration of transgenic DNA in the 3′ end of *A. tumefaciens* transformants (Hwang et al., 2017) such as canola, corresponding to plasmid cloning vector (CV) and an ExV to increase the plasmid protein expression level, were also identified in this study. In clover, the presence of large amounts of phenols, which suffer a rapid oxidation and form reactive quinones that irreversibly bind to proteins affecting their solubility (Whitlock et al., 2008), may have affected the quality of genomic DNA generating shorter reads. Despite these issues with DNA quality, the read length was sufficient to characterize a complex integration of the multi-trait construct, which was initially thought to be 9.4 kb. However, following full characterization of the insertion the transgene was identified to be 18.6 kbp in length, due to a linear concatenated insertion. In ryegrass, a fragment of plasmid plant ExV was identified in the 3′ sequence that was used in the transformation process to allow the transcription of the cloned gene. The generation of longer sequence reads greater than the transgenic insertion is highly important for the description of an event, and for any application of the approach described here, DNA quality and integrity is of the highest value. Additionally, the inclusion of all intended and unintended elements (inserted construct and plasmid sequence) in the data analysis is of high value to fully characterize the transgenic insertion.

In complex GM crop genomes, such as the examples used in the present study, both walking methods coupled with SGS and whole genome sequencing approaches are ineffective to fully characterize the transgenic insertion and flanking sequences. The size limitation of enrichment methods and assembly issues of short-read sequencing would make the characterization of plants used in this study highly challenging, due to difficulties enriching cisgenic cassettes and assembling large repeat structures with high sequence identity. In ryegrass, for instance, repetitive sequences are usually longer than the length of an SGS read, so a single short read will not completely span a repetitive region (Senol-Cali et al., 2019). Additionally, the cisgenic nature, size, and complexity of a multi-trait construct such as GM clover makes its molecular characterization with PCR enrichment and/or short-read sequencing approaches practically impossible.

The use of nanopore sequencing allowed us to obtain flanking endogenous sequences between 3.6 and 38.6 kbp and identify their exact chromosomal location and/or gene interruptions, when a reference genome was available. Furthermore, the proposed method allows the identification of undesired insertions commonly present in transgenic events. A successful application of the proposed workflow would depend on the availability of full genomes to identify the location of the insertion site, something that still represents a bottleneck. However, when a reference genome is not available, close relatives are extremely valuable. In the instances where only draft scaffolds are available, it is still possible to draw conclusions from the data generated. With the continual improvement of TGS technology, there are also an increasing number of species with significant reference genomic sequence resources.

Additionally, the application of ONT sequencing for molecular characterization of GM plants would depend on the successful establishment of bioinformatic pipelines. Fraiture et al. (2018) found challenges in the analysis of raw nanopore sequencing data to detect unauthorized GMOs, possibly as the study integrated DNA walking strategies with nanopore sequencing, which required the development of adequate pipelines, reliable bioinformatic tools, and limited the sequence length they obtained around the transgene. An advantage of the workflow proposed in this study is that there is no need of assembly, since assemblies create unavoidable assumptions made on the overlapping sequences. Although nanopore sequencing basecalling has significantly improved, generating highly accurate consensus sequences (Krehenwinkel et al., 2019), the error rate can limit its use in identifying targeted deletions or modifications typically generated in genome editing. Additionally, the bioinformatic step represents a great challenge, especially for researchers with little or no bioinformatic capability. In this study, the data analysis uses a simple strategy based on common BLAST tools, which can be used by researchers without extensive bioinformatics expertise.

## Conclusion

The aim of this study was to develop a simple, fast, and cost-effective method for molecular characterization of transgenic plants, with a simple and robust bioinformatic pipeline and strategies that would be broadly applicable and accessible. The proposed workflow can be performed in 1 week, using a single nanopore flowcell per transgenic event, and has been initially exemplified across a range of crop genome sizes up to 2.7 gb. The method described is broadly applicable for the vast majority of plant species and transgene insertions. The methodology can be used to fully characterize transgenic events at the molecular level before the commercialization or more detailed deregulation process begins, as it provides the insertion description in a short period of time. Additionally, it could potentially be used for traceability purposes, using a custom database to screen for vectors and common transgenic elements. To the best of our knowledge, the present study is the first assessment and full molecular characterization of transgenic plants using only nanopore sequencing. The approach described here will complement the range of methods for molecular characterization of GM plants, since it can make the process more cost-effective, rapid, and simple.

## Data Availability Statement

The raw data supporting the conclusions of this article will be made available by the authors, without undue reservation.

## Author Contributions

PG performed the all laboratory experimental work with guidance from HS, performed the data analysis with assistance by NC, and prepared the manuscript under the supervision and drafting of NC, HS, and KS. NC, KS, HS, and GS edited the manuscript finally. All the authors contributed to the article and approved the submitted version.

## Conflict of Interest

The authors declare that the research was conducted in the absence of any commercial or financial relationships that could be construed as a potential conflict of interest.

## Footnotes

1 https://github.com/jarekbryk/localblast

2 https://github.com/lh3/seqtk

3 www.ensembl.org

## Supplementary Material

Supplementary Figure 1 Schematic diagram of vectors used to construct the transgenic lines. **(A)** Engineered transgene integration platform (ETIP) cassette for expression in canola. **(B)** pGRA000120 cassette with the glutamate receptor for expression in ryegrass. **(C)** pCLV000032 cassette with a multi-trait construct in the left and the pDOI000080 cassette for hygromycin phosphotransferase gene (*hph*) as a selectable marker in the right for expression in clover.

Supplementary Figure 2 MinION real-time basecalling evaluation after 24 h of sequencing, using the MinKNOW software. Data acquisition and real-time analysis for **(A)** canola, **(B)** clover, and **(C)** ryegrass.

Supplementary Figure 3 Nanoplot illustration of the read lengths vs. average read quality of **(A)** canola, **(B)** clover, and **(C)** ryegrass.

Supplementary Table 1 Nucleotide BLAST results of the transgenic sequences for canola, clover, and ryegrass.
