# Supplementary figures and images for: Rapid and Detailed Characterization of Transgene Insertion Sites in Genetically Modified Plants via Nanopore Sequencing
Source: Front Plant Sci. 2021 Feb 4;11:602313. doi: 10.3389/fpls.2020.602313 (PMC7889508; doi:10.3389/fpls.2020.602313)

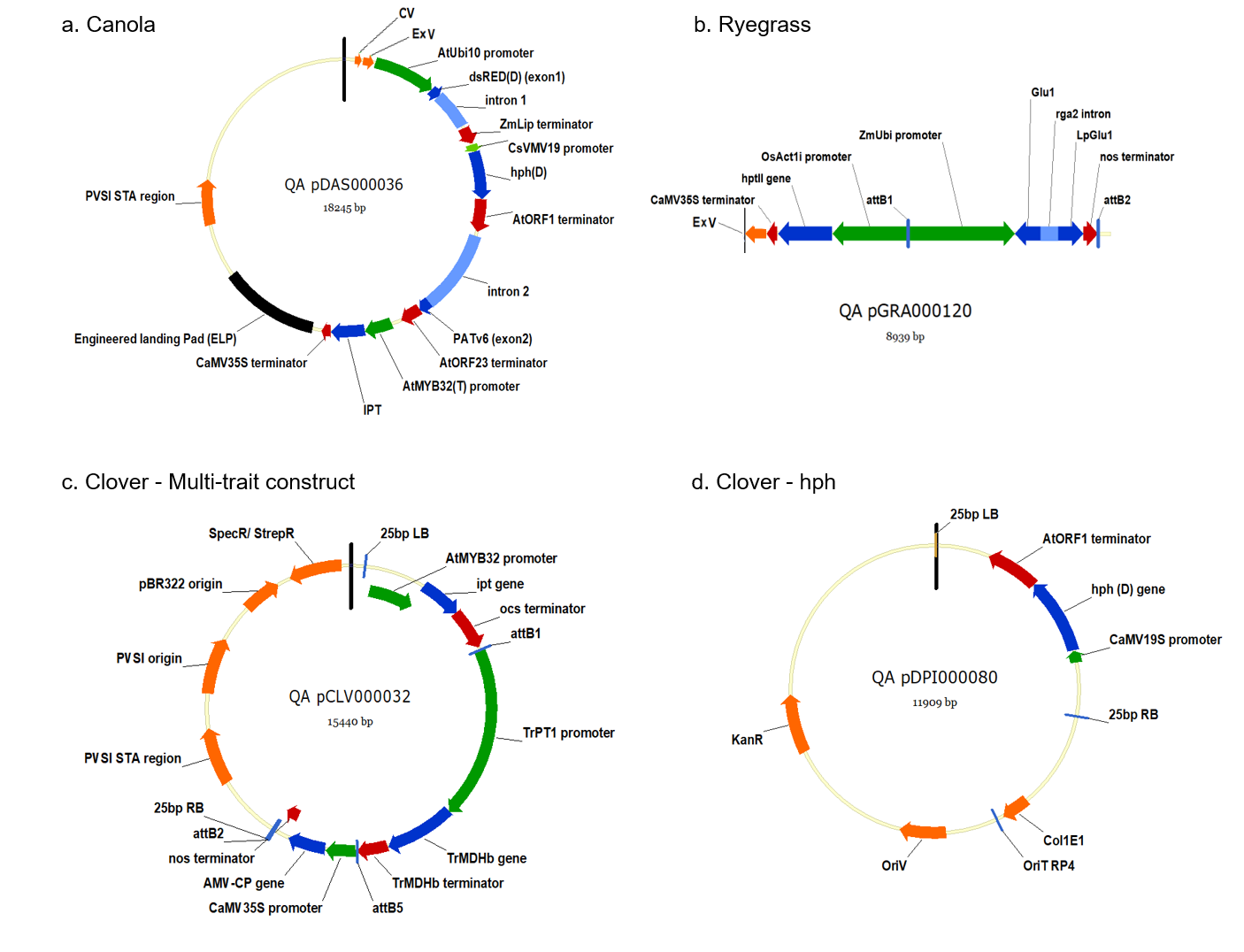

Supplement: Supplementary Figure 1 — Schematic diagram of vectors used to construct the transgenic lines. (A) Engineered transgene integration platform (ETIP) cassette for expression in canola. (B) pGRA000120 cassette with the glutamate receptor for expression in ryegrass. (C) pCLV000032 cassette with a multi-trait construct in the left and the pDOI000080 cassette for hygromycin phosphotransferase gene (hph) as a selectable marker in the right for expression in clover. [file Image_1.png]

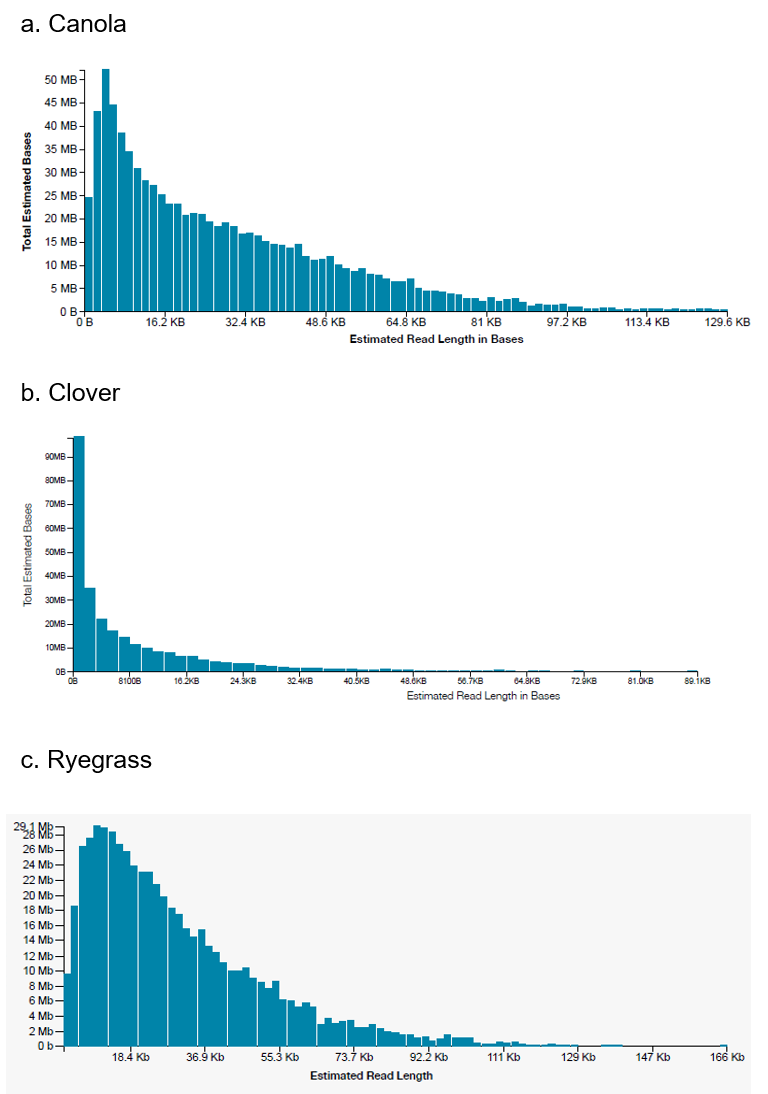

Supplement: Supplementary Figure 2 — MinION real-time basecalling evaluation after 24 h of sequencing, using the MinKNOW software. Data acquisition and real-time analysis for (A) canola, (B) clover, and (C) ryegrass. [file Image_2.png]

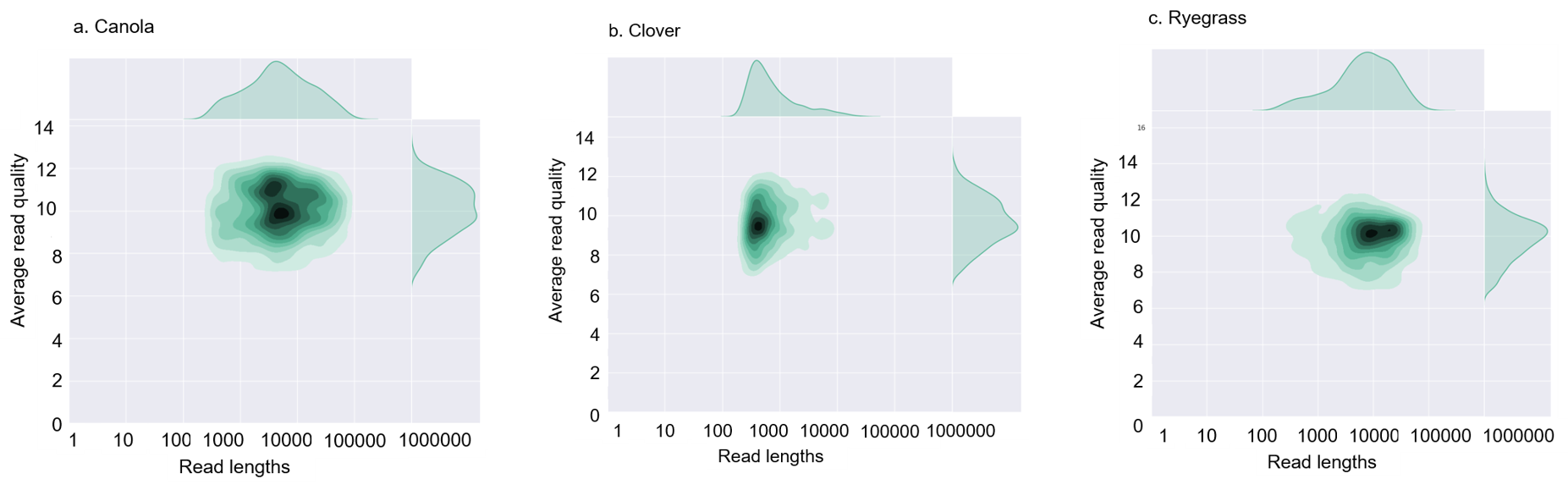

Supplement: Supplementary Figure 3 — Nanoplot illustration of the read lengths vs. average read quality of (A) canola, (B) clover, and (C) ryegrass. [file Image_3.png]
