# Supplementary material for: Rapid and Detailed Characterization of Transgene Insertion Sites in Genetically Modified Plants via Nanopore Sequencing
Source: Front Plant Sci. 2021 Feb 4;11:602313. doi: 10.3389/fpls.2020.602313 (PMC7889508; doi:10.3389/fpls.2020.602313)
Supplement: Supplementary Table 1 — Nucleotide BLAST results of the transgenic sequences for canola, clover, and ryegrass. [file Table_1.docx]

Table S1:

| **Sample** | **Query^1^** | **Subject_DB_hit^2^** | **%_ident^3^** | **Length^4^** | **Mismatch** | **Gapopen^5^** | **q_start^6^** | **q_end^7^** | **s_start^8^** | **s_end^9^** | **Evalue^10^** | **Score^11^** |
| --- | --- | --- | --- | --- | --- | --- | --- | --- | --- | --- | --- | --- |
| Canola | 0d105257-8f90-4252-90b8-273135b71fe2 | QA_pDAS000036 | 91.52 | 1757 | 49 | 71 | 30 | 1,722 | 1,721 | 1 | 0 | 2327 |
|  | 20af5b95-77aa-40be-adf9-ee4a64ba694b | QA_pDAS000036 | 90.204 | 6615 | 232 | 304 | 51,856 | 58,211 | 11,191 | 4,734 | 0 | 8242 |
|  | 20af5b95-77aa-40be-adf9-ee4a64ba694b | QA_pDAS000036 | 93.952 | 4795 | 84 | 143 | 69,049 | 73,750 | 4,682 | 1 | 0 | 7059 |
|  | 20af5b95-77aa-40be-adf9-ee4a64ba694b | QA_pDAS000036 | 91.336 | 831 | 22 | 36 | 66,795 | 67,605 | 2,337 | 1,537 | 0 | 1090 |
|  | addc2375-9275-4227-9e95-19eae56fd97a | QA_pDAS000036 | 89.881 | 3182 | 125 | 151 | 4,366 | 7,454 | 2 | 3,079 | 0 | 3910 |
|  | 31304c5d-4995-4eb2-9ca0-265db3a99100 | QA_pDAS000036 | 90.982 | 4857 | 139 | 211 | 2,051 | 6,775 | 1 | 4,690 | 0 | 6266 |
|  | 31304c5d-4995-4eb2-9ca0-265db3a99100 | QA_pDAS000036 | 88.171 | 3635 | 157 | 199 | 17,579 | 21,081 | 4,734 | 8,227 | 0 | 4078 |
|  | 31304c5d-4995-4eb2-9ca0-265db3a99100 | QA_pDAS000036 | 84.495 | 832 | 33 | 60 | 8,238 | 9,009 | 1,538 | 2,333 | 0 | 734 |
|  | 31304c5d-4995-4eb2-9ca0-265db3a99100 | QA_pDAS000036 | 91.513 | 542 | 11 | 22 | 12,764 | 13,299 | 1,537 | 2,049 | 0 | 713 |
|  | bb4c1c23-58df-496c-8512-707a62e0f559 | QA_pDAS000036 | 92.166 | 651 | 18 | 28 | 3,488 | 4,122 | 5,614 | 4,981 | 0 | 889 |
|  | 465f41de-bfc0-4f71-87c0-c36f0557db4d | QA_pDAS000036 | 92.821 | 4694 | 125 | 159 | 30 | 4,619 | 6,604 | 11,189 | 0 | 6606 |
|  | a865e4a8-a13e-48ef-812f-49af1d69b5a7 | QA_pDAS000036 | 82.95 | 1695 | 154 | 117 | 31 | 1,658 | 5,713 | 7,339 | 0 | 1404 |
|  | 3e9f64ab-d6c8-4f54-a664-2e7f67c5b820 | QA_pDAS000036 | 90.579 | 11591 | 388 | 514 | 19,021 | 30,313 | 1 | 11,185 | 0 | 14705 |
|  | e7d89412-698e-4b83-a8c1-40d10b47d71c | QA_pDAS000036 | 91.241 | 1233 | 44 | 53 | 32 | 1,228 | 9,987 | 11,191 | 0 | 1620 |
|  | fcd63137-1a34-4302-b57a-6db5a52744ac | QA_pDAS000036 | 90.49 | 673 | 28 | 30 | 14,560 | 15,223 | 5,614 | 4,969 | 0 | 856 |
|  | 88826fa1-528c-4a66-bdbf-3107c1bab87e | QA_pDAS000036 | 92.988 | 656 | 15 | 25 | 29,833 | 30,467 | 4,969 | 5,614 | 0 | 928 |
|  | 748a2b02-c2a8-4a8d-a1cf-66f7897c517a | QA_pDAS000036 | 89.117 | 6671 | 294 | 317 | 24,898 | 31,348 | 11,189 | 4,731 | 0 | 7899 |
|  | 748a2b02-c2a8-4a8d-a1cf-66f7897c517a | QA_pDAS000036 | 92.12 | 4797 | 125 | 180 | 42,238 | 46,896 | 4,682 | 1 | 0 | 6530 |
|  | 748a2b02-c2a8-4a8d-a1cf-66f7897c517a | QA_pDAS000036 | 91.849 | 822 | 30 | 27 | 39,976 | 40,781 | 2,337 | 1,537 | 0 | 1112 |
|  | 748a2b02-c2a8-4a8d-a1cf-66f7897c517a | QA_pDAS000036 | 94.268 | 471 | 14 | 10 | 39,042 | 39,508 | 7,233 | 6,772 | 0 | 708 |
|  | 0f437825-14de-4660-b48f-de0ae9f1c629 | QA_pDAS000036 | 91.765 | 680 | 10 | 31 | 34,773 | 35,440 | 4,969 | 5,614 | 0 | 904 |
|  | fd997ad1-1ed9-491e-ac9c-ec7681439035 | QA_pDAS000036 | 92.081 | 6617 | 192 | 252 | 5,075 | 11,520 | 4,734 | 11,189 | 0 | 9010 |
|  | fd997ad1-1ed9-491e-ac9c-ec7681439035 | QA_pDAS000036 | 92.176 | 524 | 18 | 14 | 308 | 825 | 1,539 | 2,045 | 0 | 719 |
|  | 7fccbd2c-3f4a-47e3-b019-c86badd61668 | QA_pDAS000036 | 90.516 | 6643 | 267 | 275 | 32,593 | 39,060 | 4,734 | 11,188 | 0 | 8442 |
|  | 7fccbd2c-3f4a-47e3-b019-c86badd61668 | QA_pDAS000036 | 93.645 | 4784 | 118 | 142 | 17,195 | 21,886 | 1 | 4,690 | 0 | 6979 |
|  | 7fccbd2c-3f4a-47e3-b019-c86badd61668 | QA_pDAS000036 | 91.799 | 817 | 19 | 32 | 23,305 | 24,089 | 1,537 | 2,337 | 0 | 1094 |
|  | 7fccbd2c-3f4a-47e3-b019-c86badd61668 | QA_pDAS000036 | 94.915 | 472 | 9 | 12 | 24,553 | 25,019 | 6,772 | 7,233 | 0 | 725 |
|  | 8efec15c-4f0f-4b22-be18-700d24dcc169 | QA_pDAS000036 | 89.11 | 2415 | 119 | 107 | 29 | 2,372 | 5,551 | 3,210 | 0 | 2870 |
|  | 6d1f8e3b-45db-44d5-9022-f7762ea4476f | QA_pDAS000036 | 90.299 | 3649 | 134 | 154 | 32 | 3,563 | 6,174 | 9,719 | 0 | 4575 |
|  | 3c4a3148-6070-4fb7-a898-143195ba26a5 | QA_pDAS000036 | 93.179 | 2419 | 51 | 87 | 11,163 | 13,520 | 4,688 | 2,323 | 0 | 3448 |
|  | 3c4a3148-6070-4fb7-a898-143195ba26a5 | QA_pDAS000036 | 89.995 | 2019 | 80 | 89 | 13,694 | 15,653 | 1,956 | 1 | 0 | 2497 |
|  | 3c4a3148-6070-4fb7-a898-143195ba26a5 | QA_pDAS000036 | 92.566 | 834 | 14 | 35 | 8,904 | 9,722 | 2,337 | 1,537 | 0 | 1153 |
|  | 3c4a3148-6070-4fb7-a898-143195ba26a5 | QA_pDAS000036 | 92.912 | 522 | 20 | 16 | 4,720 | 5,233 | 2,049 | 1,537 | 0 | 743 |
|  | b03dd5d9-b38b-410a-89bd-085039c7ae70 | QA_pDAS000036 | 91.68 | 1839 | 54 | 74 | 8,422 | 10,224 | 11,189 | 9,414 | 0 | 2457 |
| Clover Multi-trait costruct | 041fc35a-8303-49e7-b59c-0496f372f555 | QA_pCLV000032 | 90.43 | 2779 | 74 | 153 | 31 | 2718 | 5731 | 3054 | 0 | 3482 |
|  | 0594f3b4-5d90-430b-837e-c64617613c4a | QA_pCLV000032 | 89.91 | 1387 | 38 | 86 | 854 | 2188 | 4480 | 5816 | 0 | 1692 |
|  | 0b0d5005-ee38-4ae5-ba80-335353f6d801 | QA_pCLV000032 | 88.6 | 3123 | 98 | 169 | 12544 | 15551 | 5813 | 2834 | 0 | 3557 |
|  | 0b0d5005-ee38-4ae5-ba80-335353f6d801 | QA_pCLV000032 | 88.64 | 1752 | 62 | 109 | 26871 | 28549 | 4521 | 2834 | 0 | 2006 |
|  | 0b0d5005-ee38-4ae5-ba80-335353f6d801 | QA_pCLV000032 | 87.01 | 1417 | 61 | 90 | 25001 | 26377 | 5813 | 4480 | 0 | 1483 |
|  | 0e019a27-291a-4f67-9bed-16d11d9d7115 | QA_pCLV000032 | 89.16 | 959 | 29 | 51 | 27 | 933 | 3568 | 4503 | 0 | 1125 |
|  | 0e897f43-f050-4867-b4ef-9d0246754204 | QA_pCLV000032 | 87.15 | 2474 | 80 | 168 | 15197 | 17604 | 5816 | 3515 | 0 | 2588 |
|  | 11dc013b-8919-4b4a-b999-796c2c01c34e | QA_pCLV000032 | 90.05 | 3317 | 87 | 194 | 31 | 3264 | 3842 | 6998 | 0 | 4072 |
|  | 14209d5e-ee1d-4328-a390-a6205db3dc19 | QA_pCLV000032 | 89.6 | 1279 | 38 | 72 | 22 | 1250 | 4842 | 6075 | 0 | 1537 |
|  | 14b4c94c-a5fa-4dbd-96e2-8bc5b163789e | QA_pCLV000032 | 82.44 | 1230 | 83 | 113 | 31 | 1184 | 9158 | 7986 | 0 | 952 |
|  | 197024cd-6fb5-4ef1-83aa-1d4787447373 | QA_pCLV000032 | 92.02 | 990 | 16 | 46 | 38 | 997 | 2182 | 3138 | 0 | 1332 |
|  | 1a71033b-c5d7-4aee-a120-861866ff7b93 | QA_pCLV000032 | 84.18 | 1801 | 82 | 153 | 860 | 2565 | 7511 | 5819 | 0 | 1559 |
|  | 1b3c1166-2fb8-4b87-917d-d4ee8ac56c35 | QA_pCLV000032 | 89.18 | 1867 | 53 | 120 | 31 | 1823 | 5745 | 7536 | 0 | 2191 |
|  | 1e9a965d-e3cc-494c-a248-5574e03a471b | QA_pCLV000032 | 92.95 | 3022 | 60 | 128 | 29 | 2973 | 3190 | 245 | 0 | 4259 |
|  | 1f994575-47ec-467e-8360-158a5b0646e3 | QA_pCLV000032 | 88.08 | 1854 | 58 | 127 | 1113 | 2900 | 5819 | 7575 | 0 | 2049 |
|  | 21c19895-6a32-41d9-a57f-8685c9f740e4 | QA_pCLV000032 | 87.36 | 1495 | 65 | 101 | 42 | 1465 | 5779 | 4338 | 0 | 1600 |
|  | 31d9b8a1-6206-49fb-a79d-617a836485fd | QA_pCLV000032 | 89.32 | 1779 | 52 | 100 | 3334 | 5060 | 7511 | 5819 | 0 | 2106 |
|  | 35475822-f938-45f7-90f0-84a6e5574b07 | QA_pCLV000032 | 89.18 | 3171 | 86 | 170 | 15586 | 18690 | 5813 | 2834 | 0 | 3718 |
|  | 4336b3d8-aef2-4e2e-9cf3-e3e999f64e3e | QA_pCLV000032 | 90.95 | 763 | 15 | 36 | 30 | 673 | 7057 | 6421 | 0 | 850 |
|  | 479af3f9-618a-4370-bbe6-847d7bc7cfa4 | QA_pCLV000032 | 89.43 | 880 | 40 | 48 | 378 | 1226 | 2834 | 3691 | 0 | 1061 |
|  | 4b0e5c96-2ba2-40bc-8e2e-27d4cd9d1b16 | QA_pCLV000032 | 90.51 | 3911 | 99 | 201 | 26912 | 30667 | 1026 | 4819 | 0 | 4916 |
|  | 4c4f2ce2-dba8-4d64-aaef-d761d2c74ed9 | QA_pCLV000032 | 90.86 | 5774 | 152 | 294 | 33 | 5642 | 7188 | 1627 | 0 | 7391 |
|  | 50def966-8658-4e15-9908-d4e9d8ff1170 | QA_pCLV000032 | 92.61 | 1096 | 21 | 48 | 29 | 1110 | 2685 | 1636 | 0 | 1520 |
|  | 55a6039d-d961-4a4c-80c0-878b66a0edef | QA_pCLV000032 | 90.4 | 1770 | 40 | 96 | 4973 | 6703 | 5816 | 4138 | 0 | 2207 |
|  | 5b74baf4-f9bb-419f-baaf-bf738c95c201 | QA_pCLV000032 | 86.69 | 3260 | 160 | 225 | 50 | 3168 | 8439 | 5313 | 0 | 3363 |
|  | 5e7e813a-d1b3-4a7c-8d14-a470fd8f4b52 | QA_pCLV000032 | 89.73 | 2221 | 51 | 125 | 36 | 2169 | 3980 | 1850 | 0 | 2675 |
|  | 600e5614-2d84-4045-8c15-3e3089772394 | QA_pCLV000032 | 90.01 | 931 | 31 | 48 | 62 | 963 | 4470 | 3573 | 0 | 1147 |
|  | 60e012b6-8e17-43ac-9ac6-7d284c109c02 | QA_pCLV000032 | 92.41 | 1753 | 29 | 76 | 1366 | 3079 | 1021 | 2708 | 0 | 2405 |
|  | 612e9f72-ecd1-423d-b452-462d509d5d0c | QA_pCLV000032 | 90.4 | 792 | 18 | 41 | 28 | 799 | 3406 | 4159 | 0 | 989 |
|  | 6604ee66-984f-47af-835c-ec5858d6715d | QA_pCLV000032 | 88.32 | 1764 | 81 | 103 | 2052 | 3767 | 7505 | 5819 | 0 | 2001 |
|  | 691d370a-fd36-4fdf-bbe6-fe0ad8d036a2 | QA_pCLV000032 | 83.87 | 1283 | 64 | 124 | 39 | 1261 | 7135 | 8334 | 0 | 1090 |
|  | 6cdd2ec8-dff1-4bae-915b-c6f75bdf4a12 | QA_pCLV000032 | 89.81 | 1443 | 44 | 81 | 31 | 1423 | 6420 | 5031 | 0 | 1755 |
|  | 71a9c389-6995-4bb0-918d-f579a25adcaf | QA_pCLV000032 | 84.59 | 3011 | 150 | 247 | 1675 | 4517 | 5760 | 8624 | 0 | 2700 |
|  | 71a9c389-6995-4bb0-918d-f579a25adcaf | QA_pCLV000032 | 87.79 | 1687 | 59 | 112 | 34 | 1642 | 4070 | 5687 | 0 | 1838 |
|  | 77d7259c-f4f6-4cf0-8082-e9515480e6b1 | QA_pCLV000032 | 79.61 | 3635 | 246 | 408 | 897 | 4285 | 12864 | 9479 | 0 | 2150 |
|  | 7dade372-5567-4024-97f0-9ff2d8b8ab8d | QA_pCLV000032 | 90.66 | 5762 | 152 | 281 | 39 | 5665 | 326 | 5836 | 0 | 7304 |
|  | 81ddc988-c7b5-42e1-9599-bb1499d2dd37 | QA_pCLV000032 | 90.79 | 1184 | 32 | 59 | 3902 | 5053 | 5819 | 6957 | 0 | 1511 |
|  | 855ffea6-9877-4b03-9836-557729154c61 | QA_pCLV000032 | 91.52 | 1368 | 29 | 68 | 31 | 1377 | 5760 | 7061 | 0 | 1803 |
|  | 87f9d285-bae4-41e8-b095-f558668ad5e2 | QA_pCLV000032 | 89.86 | 769 | 17 | 46 | 30 | 772 | 3576 | 2843 | 0 | 931 |
|  | 89604cfc-ce44-4d72-af53-f227a1ab9f5a | QA_pCLV000032 | 90.89 | 1504 | 40 | 73 | 5147 | 6597 | 2711 | 1252 | 0 | 1929 |
|  | 91ea965e-b37b-41a0-98e8-0783799f0c6f | QA_pCLV000032 | 87.78 | 1146 | 38 | 70 | 35 | 1153 | 3591 | 2521 | 0 | 1247 |
|  | 9abab516-486b-45ea-a993-d48a6b75dabd | QA_pCLV000032 | 86.98 | 1536 | 62 | 117 | 29 | 1496 | 7284 | 5819 | 0 | 1602 |
|  | a446bf30-79fd-4d59-a843-f467c5ececc8 | QA_pCLV000032 | 87.64 | 1755 | 72 | 113 | 706 | 2383 | 5825 | 7511 | 0 | 1905 |
|  | a7ce9cad-0223-41f0-a805-444ee7ec6655 | QA_pCLV000032 | 91.91 | 1174 | 23 | 61 | 16229 | 17370 | 5816 | 4683 | 0 | 1576 |
|  | adbe0b0c-4f22-4fa0-8d20-2518289dc74b | QA_pCLV000032 | 86.51 | 1045 | 42 | 77 | 80 | 1097 | 3527 | 4499 | 0 | 1057 |
|  | adbe0b0c-4f22-4fa0-8d20-2518289dc74b | QA_pCLV000032 | 87.94 | 945 | 36 | 60 | 1120 | 2050 | 4484 | 5364 | 0 | 1042 |
|  | adc9dbd2-7393-42f2-a1af-4a8bcc437ad4 | QA_pCLV000032 | 89.49 | 6876 | 222 | 405 | 1395 | 8033 | 2508 | 9119 | 0 | 8229 |
|  | ade6ccbc-f56f-4f1c-81c9-aedfa0912041 | QA_pCLV000032 | 91.11 | 1114 | 26 | 55 | 40 | 1119 | 2336 | 1262 | 0 | 1441 |
|  | b19b889f-354b-4ad2-9e23-b4beead9dcf2 | QA_pCLV000032 | 93.8 | 710 | 9 | 26 | 33 | 723 | 6427 | 7120 | 0 | 1035 |
|  | b301af45-a8dd-4e59-9e70-24eee5ab87dd | QA_pCLV000032 | 92.8 | 1751 | 37 | 69 | 1425 | 3140 | 1013 | 2709 | 0 | 2453 |
|  | b4e1c684-45d6-4475-9935-c3f7ccc9ef58 | QA_pCLV000032 | 87.95 | 772 | 24 | 58 | 31 | 764 | 6490 | 7230 | 0 | 846 |
|  | bce8fd5d-441b-4ef4-8e4e-3f9c08a5dc41 | QA_pCLV000032 | 92.3 | 2065 | 37 | 81 | 30 | 2050 | 3031 | 1045 | 0 | 2820 |
|  | c143c0b9-dbb8-4441-84a3-60310f01170d | QA_pCLV000032 | 90.72 | 1767 | 42 | 86 | 7435 | 9165 | 7499 | 5819 | 0 | 2242 |
|  | c6498baf-d958-4866-a98a-456e854ff109 | QA_pCLV000032 | 92.15 | 1044 | 28 | 43 | 38 | 1048 | 2283 | 3305 | 0 | 1424 |
|  | cd274e7f-82d2-47cc-b748-01947d7a0d36 | QA_pCLV000032 | 92.16 | 969 | 20 | 42 | 31 | 982 | 3250 | 4179 | 0 | 1317 |
|  | cfbd819a-541f-4fea-afeb-7163ae7d1e9f | QA_pCLV000032 | 88.55 | 2402 | 65 | 141 | 29 | 2334 | 3526 | 5813 | 0 | 2719 |
|  | d110722a-a946-401a-912c-2a0c332ed648 | QA_pCLV000032 | 80.41 | 1756 | 109 | 191 | 220 | 1868 | 13802 | 15429 | 0 | 1120 |
|  | d110722a-a946-401a-912c-2a0c332ed648 | QA_pCLV000032 | 89.2 | 741 | 17 | 50 | 1894 | 2604 | 9 | 716 | 0 | 867 |
|  | d330f17a-5f6a-4a4c-aa53-4c33a14bc76a | QA_pCLV000032 | 85.28 | 4750 | 216 | 372 | 34 | 4513 | 4537 | 1 | 0 | 4453 |
|  | d330f17a-5f6a-4a4c-aa53-4c33a14bc76a | QA_pCLV000032 | 82.88 | 4475 | 278 | 382 | 10531 | 14781 | 8281 | 4071 | 0 | 3570 |
|  | d476bb30-c2e3-4324-b609-32a5d9992806 | QA_pCLV000032 | 84.06 | 853 | 37 | 76 | 30 | 822 | 6496 | 7309 | 0 | 730 |
|  | d887ab8e-1e24-4616-a8de-71d117931a0b | QA_pCLV000032 | 86.62 | 2518 | 114 | 172 | 30 | 2430 | 3402 | 5813 | 0 | 2577 |
|  | e260fb29-1e89-430a-8121-09c48558e02e | QA_pCLV000032 | 92.16 | 1747 | 44 | 62 | 1111 | 2812 | 2711 | 1013 | 0 | 2381 |
|  | f000de16-da27-4fa5-8dd3-e88424c2dde1 | QA_pCLV000032 | 85.7 | 1280 | 43 | 112 | 31 | 1251 | 8274 | 7076 | 0 | 1221 |
|  | fffb1347-eb1c-4e69-b2fe-8f347a4ed277 | QA_pCLV000032 | 90.49 | 1472 | 57 | 72 | 32 | 1466 | 6156 | 7581 | 0 | 1866 |
| Clover hph | 06218884-6f57-4c4a-9713-6e3590acfe25 | QA_pDPI000080 | 84.54 | 3686 | 177 | 293 | 32 | 3515 | 8228 | 11722 | 0 | 3286 |
|  | 08e0fd90-ffe8-44a0-a885-0cacd85abf5f | QA_pDPI000080 | 82.07 | 2225 | 135 | 204 | 787 | 2846 | 5904 | 8029 | 0 | 1655 |
|  | 0cf9ee31-d92a-465b-ba86-2361ef0bf09b | QA_pDPI000080 | 78.53 | 2119 | 173 | 235 | 1398 | 3361 | 678 | 2669 | 0 | 1133 |
|  | 24d4d89c-5471-400f-aa8e-e3d479bea474 | QA_pDPI000080 | 90.97 | 997 | 24 | 50 | 29 | 996 | 2330 | 1371 | 0 | 1282 |
|  | 2eda94a1-9046-4013-aa55-25badc1ec24f | QA_pDPI000080 | 89.32 | 1329 | 39 | 81 | 34 | 1325 | 1319 | 2581 | 0 | 1572 |
|  | 6a3493e9-f547-4ce0-902c-a5ea451a823e | QA_pDPI000080 | 91.52 | 2087 | 60 | 96 | 2992 | 5019 | 641 | 2669 | 0 | 2765 |
|  | 76d3764e-b965-4e2e-b542-41456ab55ae7 | QA_pDPI000080 | 86.11 | 1267 | 60 | 88 | 30 | 1251 | 1248 | 53 | 0 | 1258 |
|  | 89604cfc-ce44-4d72-af53-f227a1ab9f5a | QA_pDPI000080 | 89.9 | 2713 | 73 | 165 | 346 | 2958 | 53 | 2664 | 0 | 3306 |
|  | 8b94ede3-1ba1-4d39-9465-649b08185dec | QA_pDPI000080 | 80.09 | 3284 | 232 | 311 | 252 | 3319 | 6590 | 9667 | 0 | 2052 |
|  | ad4a3421-a705-497d-bd85-c8cc1644d481 | QA_pDPI000080 | 83.06 | 1517 | 92 | 135 | 101 | 1531 | 534 | 1971 | 0 | 1225 |
|  | da7dc82e-7a43-4e60-b575-7b2169d90cde | QA_pDPI000080 | 80.02 | 2833 | 205 | 281 | 1154 | 3847 | 11725 | 9115 | 0 | 1762 |
|  | e25de309-008e-462d-89a5-9be68438b68c | QA_pDPI000080 | 92.83 | 2107 | 38 | 92 | 3537 | 5608 | 641 | 2669 | 0 | 2950 |
|  | e7acc702-1e68-458d-91c9-aa81602d46e4 | QA_pDPI000080 | 87.71 | 1245 | 35 | 87 | 3177 | 4371 | 6637 | 7813 | 0 | 1343 |
|  | fa8ef48a-8dd6-4e00-9488-c1591f5ca1f0 | QA_pDPI000080 | 88.99 | 1017 | 28 | 58 | 57 | 1055 | 7907 | 6957 | 0 | 1181 |
| Ryegrass | 0ed1a91b-e217-4ea4-8568-d04f7f99abb8 | pGRA000120 | 89.803 | 5992 | 254 | 272 | 10962 | 16747 | 1 | 5841 | 0 | 7350 |
|  | 0ed1a91b-e217-4ea4-8568-d04f7f99abb8 | pGRA000120 | 87.711 | 1302 | 67 | 62 | 7270 | 8502 | 1196 | 2473 | 0 | 1432 |
|  | 0fafd0d7-c15d-44e3-93dc-8a836d739530 | pGRA000120 | 90.223 | 2240 | 83 | 100 | 25 | 2169 | 2199 | 1 | 0 | 2798 |
|  | 27873966-2702-46dc-a467-254f49da6548 | pGRA000120 | 91.549 | 497 | 18 | 16 | 15541 | 16024 | 5481 | 5966 | 0 | 664 |
|  | 27873966-2702-46dc-a467-254f49da6548 | pGRA000120 | 91.515 | 498 | 18 | 16 | 15543 | 16024 | 5158 | 4675 | 0 | 660 |
|  | 7cdea3d1-1a43-46d0-9cba-50adce66d12e | pGRA000120 | 86.158 | 997 | 45 | 70 | 56969 | 57907 | 1634 | 673 | 0 | 990 |
|  | 7d948122-5e5d-4e08-80aa-6b6b3a811fbe | pGRA000120 | 84.584 | 1466 | 77 | 117 | 1887 | 3242 | 208 | 1634 | 0 | 1317 |
|  | 9e717a06-501d-49a2-8f6f-e6c4d773f75b | pGRA000120 | 80.652 | 2667 | 252 | 191 | 3345 | 5850 | 5367 | 2804 | 0 | 1823 |

^1^ Query: the sequence you input into BLAST, ^2^ Subject_DB_hit: the reference sequence from your BLAST database that matched the query, ^3^ %_ident: percent identity, ^4^ Length: alignment length, ^5^ Gapopen: number of gap openings, ^6^ q_start: first query nucleotide, ^7^ q_end: last query nucleotide ^8^ s_start: first subject nucleotide ^9^ s_end: last subject nucleotide ^10^ Evalue: number of expected hits of similar quality (score) that could be found just by chance ^11^ Score: scoring local ungapped alignments.
